# A Preliminary Investigation of General and Technique-specific Assessments for the Evaluation of Laparoscopic Technical Skills

**DOI:** 10.7759/cureus.1757

**Published:** 2017-10-07

**Authors:** Ashley Vergis, Sarah Steigerwald

**Affiliations:** 1 Surgery, University of Manitoba

**Keywords:** assessment tool, laparoscopic surgery, technical skills, medical education

## Abstract

Background

Both general and technique-specific assessments of technical skill have been validated in surgical education. The purpose of this study was to assess the correlation between the objective structured assessment of technical skills (OSATS) and the global operative assessment of laparoscopic skills (GOALS) rating scales using a high-fidelity porcine laparoscopic cholecystectomy model.

Methods

Post-graduate year-one general surgery and urology residents (n=14) performed a live laparoscopic porcine cholecystectomy. Trained surgeons rated their performance using OSATS and GOALS assessment scales.

Results

Pearson’s correlation coefficient between OSATS and GOALS was 0.96 for overall scores. It ranged from 0.78 - 0.89 for domains that overlapped between the two scales.

Conclusion

There is a very high correlation between OSATS and GOALS. This implies that they likely measure similar constructs and that either may be used for summative-type assessments of trainee skill. However, further investigation is needed to determine if technique-specific assessments may provide more useful feedback in formative evaluation.

## Introduction

Valid and reliable methods of assessing technical performance are essential for surgical training programs and educational research [1]. They afford the maintenance of academic standards and function to provide feedback to learners as they progress through training.

**Figure 1:**
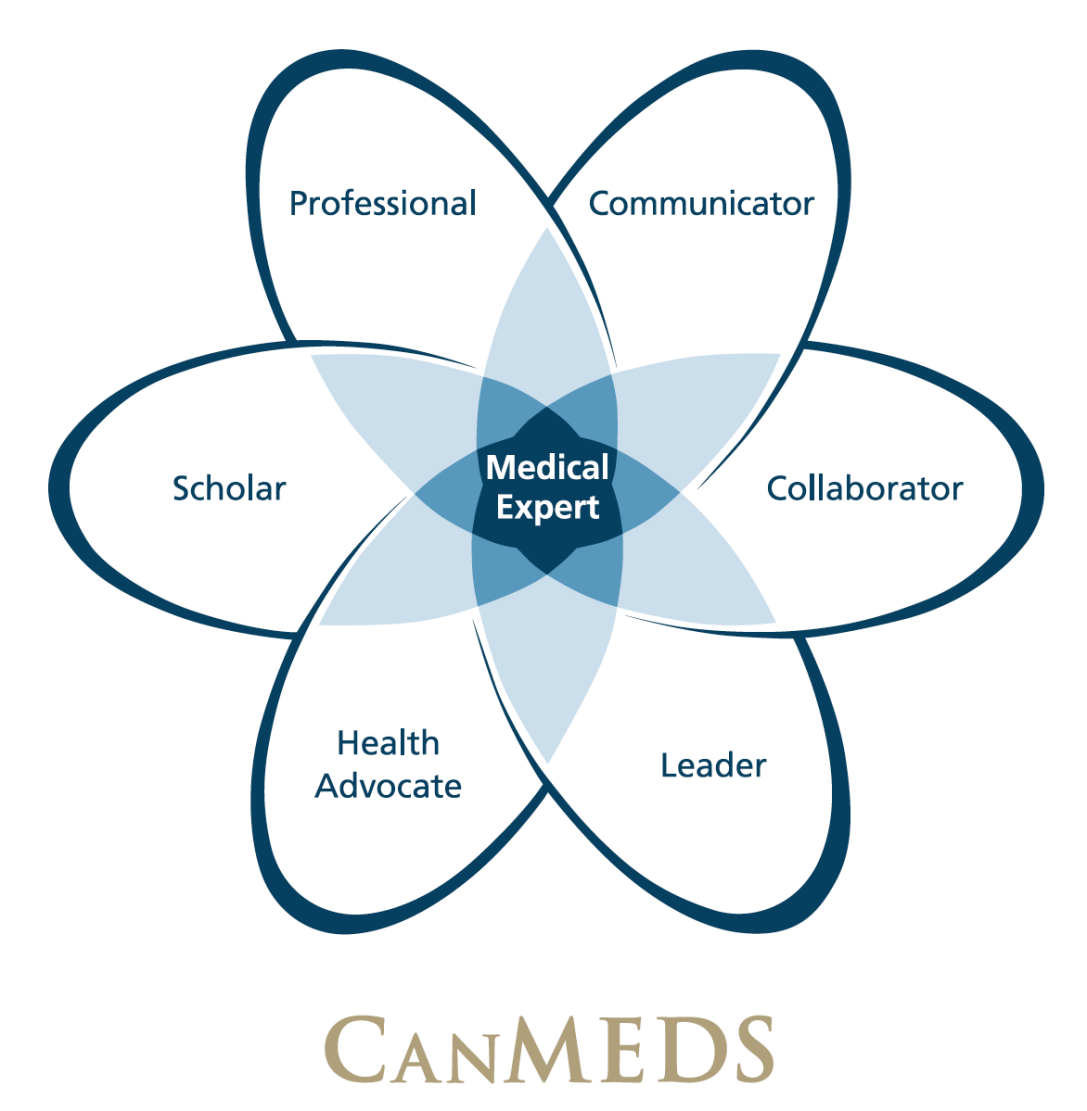
CanMEDS Framework Copyright © 2015 The Royal College of Physicians and Surgeons of Canada. Reproduced with permission. CanMEDS: Canadian Medical Education Directions for Specialists

Surgical skills are most commonly assessed using in-training evaluation reports (ITERs) in Canada. These are composed of rating scales designed to assess the Canadian Medical Education Directions for Specialists (CanMEDS) competencies in addition to technical skills (Figure 1) [2]. However, criticisms of using ITERs to evaluate technical skills include poor validity and limited inter and intra-rater reliability [3-5].

In response, many methods have been developed to objectively measure technical skills. Two important methods in laparoscopic general surgery include the objective structured assessment of technical skills (OSATS) and the global operative assessment of laparoscopic skills (GOALS) global rating scales [6]. Each consists of domains that encompass aspects of operative performance (OSATS n=7, maximum score 35; GOALS n=5, maximum score 25) that are anchored on a 5-point Likert scale. OSATS and GOALS have been shown to be valid and reliable for bench and operative settings in multiple investigations [6-17].

**Figure 2:**
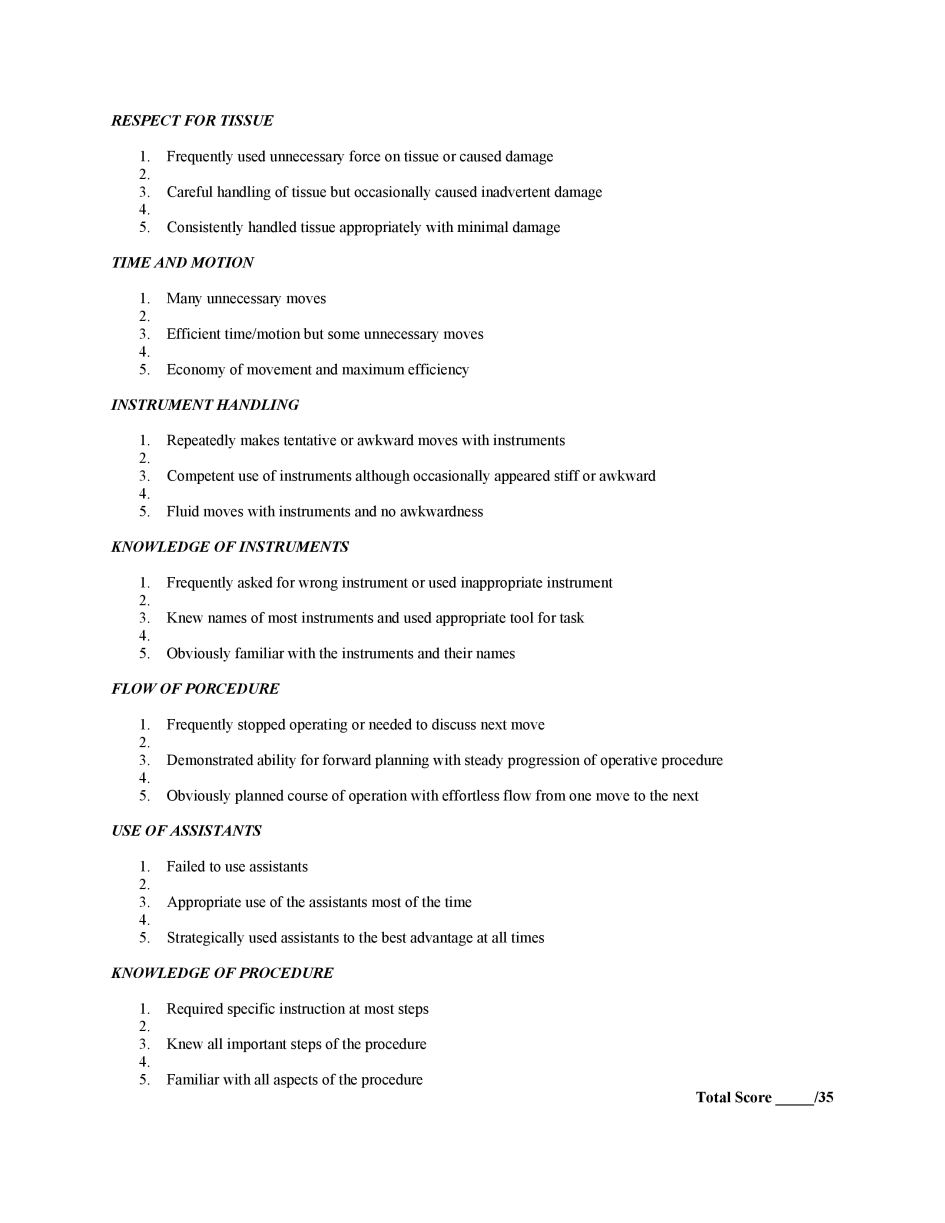
Objective Structured Assessment of Technical Skills (OSATS)

OSATS was initially developed as a general performance-based, bench-model examination consisting of eight 15-minute bench-model stations but has moved from use in the laboratory setting to use in the operating theatre (Figure 2).

**Figure 3:**
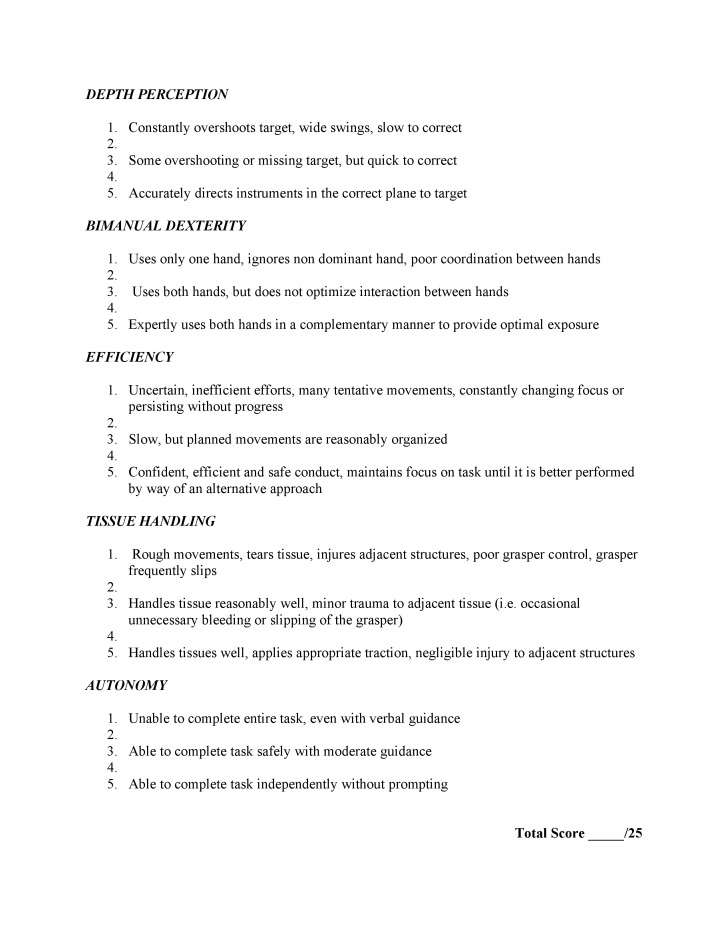
Global Operative Assessment of Laparoscopic Skills (GOALS)

GOALS is a technique-specific tool developed to specifically assess operative skills in laparoscopic surgery (Figure 3). The assumption is that laparoscopic surgery requires a more specialized assessment due to the unique, technique-specific skills associated with it. These include depth perception through a limited two-dimensional viewing field and bimanual dexterity with laparoscopic instruments. However, the presence of multiple, validated-forms of assessment calls into question the need for both general and technique-specific assessments of technical skills in surgical education.

The purpose of this investigation is to evaluate the correlation between the OSATS and the GOALS rating scales using a live-porcine laparoscopic cholecystectomy model in a summative fashion.

## Materials and methods

Fourteen postgraduate, year-one general surgery (n=11) and urology (n=3) residents at the University of Manitoba participated in this study during their surgical skills sessions. This number represents all of the trainees registered in these sessions during the study period. The residents each performed a cholecystectomy using a live porcine animal model. Prior to this, they were given didactic instruction on relevant equipment setup and use, relevant anatomy, and procedural steps.

Each performance was evaluated using OSATS and GOALS global rating scales by one of two faculty evaluators. Both evaluators were academic laparoscopic surgeons with extensive experience and training in using each rating scale. Additionally, both evaluators participated in the external validation of GOALS. We were only able to have one surgeon present at each performance due to logistical constraints. Each surgeon evaluated one-half of the participants. The University of Manitoba Health Ethics Research Board granted ethical approval to carry out the study by written consent (HS13026). The University of Manitoba Bannatyne Campus Animal Protocol Managment and Review Committee approved the use of animals in this study (15-020).

Data analysis

Correlation between the OSATS and GOALS rating scales was examined using Pearson’s coefficient, IBM Statistical Package for Social Sciences (SPSS), Version 20.0 (IBM Corp., Armonk, NY). This was carried out for overall scores and overlapping domains on the two scales.

## Results

Mean (overall) and overlapping individual OSATS and GOALS domains are presented in Table 1.

**Table 1:** Mean score (range) of overall and overlapping individual OSATS and GOALS domains OSATS: Objective Structured Assessment of Technical Skills GOALS: Global Operative Assessment of Laparoscopic Skills

	OSATS /35	GOALS /25
Overall	16.8 (9-26), 48% (25.7%-74.3%)	12.4 (7-19), 49.6% (28%-76%)
Respect for tissue/tissue handling	2.6 (1-5)	2.7 (1-5)
Time and motion/efficiency	2.1 (1-4)	2.3 (1-4)
Instrument Handling/Bi manual dexterity	2.4 (1-4)	2.3 (1-4)

**Figure 4:**
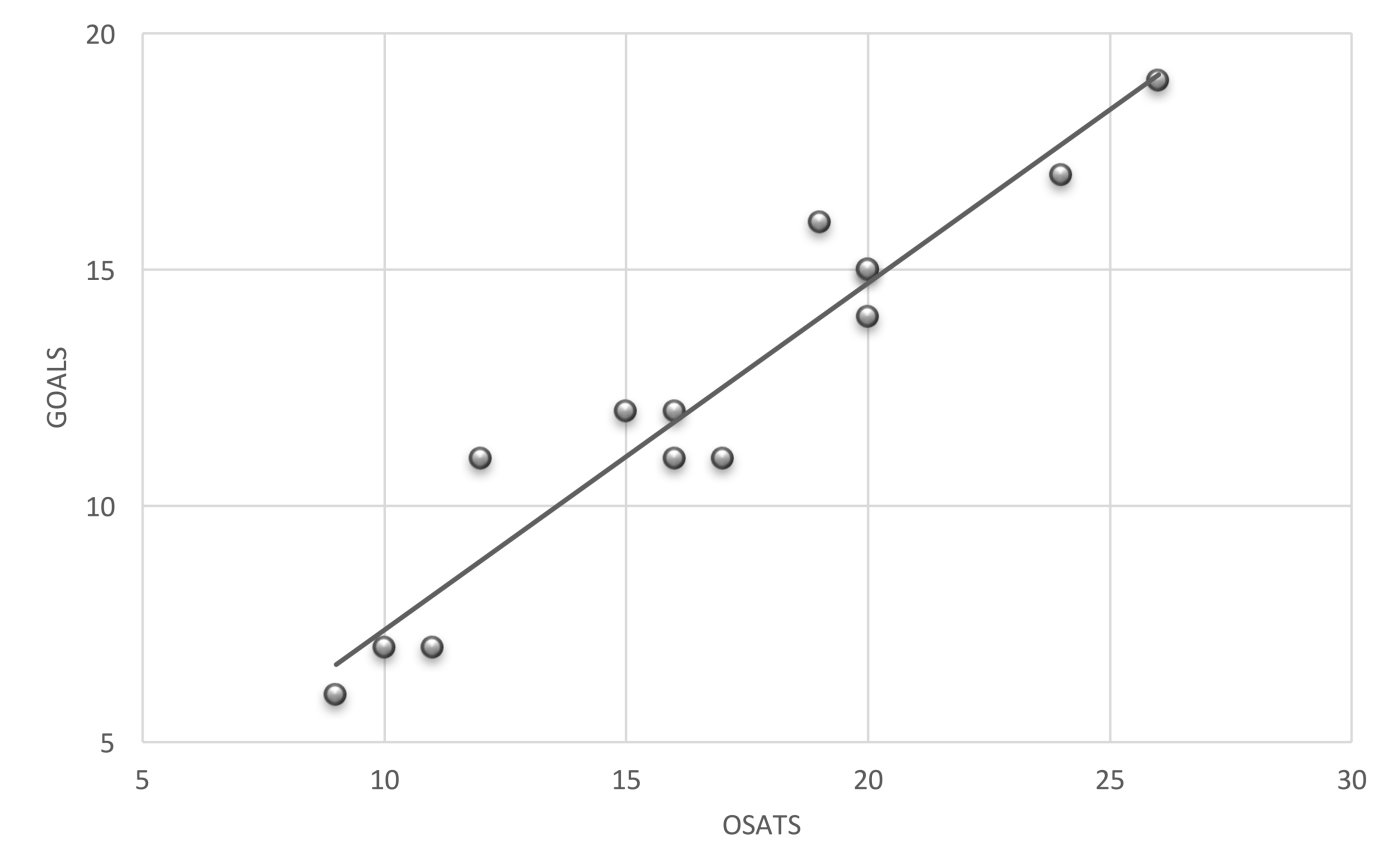
Pearson's correlation coefficient between participants OSATS and GOALS scores, R2=0.92 Note : 2 of 14 participants had identical scores and are seen only as one data point (OSATS = 20, GOALS = 15) OSATS: Objective Structured Assessment of Technical Skills GOALS: Global Operative Assessment of Laparoscopic Skills

A high correlation was demonstrated between overall OSATS and GOALS assessments with a Pearson’s correlation coefficient of 0.96 (p=0.01) (Figure 4).

Each overlapping domain also demonstrated high correlation (range 0.78-0.89, p<0.05, Table 2.)

**Table 2:** Pearson’s Correlation for overlapping OSATS and GOALS domains OSATS: Objective Structured Assessment of Technical Skills GOALS: Global Operative Assessment of Laparoscopic Skills

Domain		
OSATS	GOALS	Pearson’s Correlation (p<0.05)
Respect for Tissue	Tissue Handling	0.85
Time and Motion	Efficiency	0.78
Instrument Handling	Bimanual Dexterity	0.89
Overall Score	Overall Score	0.96

## Discussion

OSATS and GOALS have been shown to be valid and reliable assessment tools for bench and operative settings in multiple investigations internationally and within our own institution [6-7, 13-14, 16-17]. In this investigation, concurrent evidence for validity was demonstrated by showing a near total correlation between the two scales’ overall scores and in their overlapping domains. A strength of this protocol is that the porcine model allowed trainees to engage in a high-fidelity in vivo operative model without attending-surgeon intervention, as may be required for safety in a human model. This allows a more accurate assessment of trainee skill, particularly in the novice cohort.

Essentially, Pearson’s correlation is a measure of overlap between different scales. High values imply that scales are measuring similar constructs, while low values imply that scales are measuring dissimilar ones. The high correlation found suggests that OSATS and GOALS assess similar domains.

This finding questions the need for a separate laparoscopic assessment form as the more general OSATS may be used as effectively for assessment of laparoscopic skills. Advantages of using a single form for assessment in surgical education are many. First, it provides a common framework for use by raters. This may allow for a more consistent nomenclature, thus standardizing assessments across surgical platforms. This may improve reliability. Second, it is not limited by surgical approaches or platforms. A single scale may be applied to open, laparoscopic, laparoscopic-assisted, laparoscopic-converted-to-open, and potentially combined laparoscopic-endoscopic procedures.

These advantages may tempt one to conclude that is better for programs to focus on OSATS as it is validated for multiple general technical skills. However, this inference does not account for other valuable aspects of trainee assessment. Namely, it does not address the importance of feedback. While it is apparent that both forms have domains that grossly overlap, the correlation does not account for performance inferences learners may glean from the more specific assessment method. For example, the GOALS domain of bimanual dexterity significantly correlates with the OSATS domain of instrument handling (R=0.89). However, the specific anchor points on GOALS for this domain (e.g., 3= “uses both hands, but does not optimize interaction between hands”) may provide more specific feedback to the learner in laparoscopic surgery than the more general OSATS assessment (e.g., 3= “competent use of instruments but occasionally appears stiff or awkward”). Thus, both tools are acceptable in making summative assessments of laparoscopic skills as they appear to measure an overarching competency-set. However, further investigation is needed to determine the degree to which technique-specific assessments afford formative feedback in the learning process.

We believe the results of this preliminary study warrants further investigation into the use of general and technique-specific assessments for evaluating laparoscopic skills in surgical education. However, this study does have limitations. These limitations include the results being drawn from a single institution with only 14 participants and using a limited number of raters. Although this number is consistent with related educational, preliminary studies, each form has known a high inter-rater agreement. A multicenter study with a larger sample size would strengthen the results.  

## Conclusions

General and technique-specific assessments of laparoscopic skill appear to map similar domains and are shown to correlate highly. This correlation has implications for their use in both summative and formative contexts.
